# Implementing digital respiratory technologies for people with respiratory conditions: A protocol for a scoping review

**DOI:** 10.1371/journal.pone.0314914

**Published:** 2024-12-27

**Authors:** Chi Yan Hui, Kathleena Condon, Shailesh Kolekar, Nicola Roberts, Katherina Bernadette Sreter, Sami O. Simons, Carlos Figueiredo, Zoe McKeough, Hani Salim, Aleksandra Gawlik-Lipinski, Apolline Gonsard, Ayşe Önal Aral, Anna Vanoverschelde, Matthew Armstrong, Dario Kohlbrenner, Cátia Paixão, Patrick Stafler, Efthymia Papadopoulou, Adrian Paul Rabe, Milan Mohammad, Izolde Bouloukaki, Shirley Quach, Malek Chaabouni, Georgios Kaltsakas, Kate Loveys, Tonje Reier-Nilsen, Anthony Paulo Sunjaya, Paul Robinson, Hilary Pinnock, Amy Hai Yan Chan

**Affiliations:** 1 Allergy and Respiratory Research Group, Usher Institute, The University of Edinburgh, Edinburgh, United Kingdom; 2 Health Research Centre, The University of Queensland, Brisbane, Queensland, Australia; 3 Department of Respiratory Medicine, Zealand University Roskilde Hospital, Institute of Clinical Medicine Copenhagen University, Copenhagen, Denmark; 4 School of Health and Social Care, Edinburgh Napier University, Edinburgh, United Kingdom; 5 Department of Pulmonology, University Hospital Centre, Zagreb, Croatia; 6 Department of Respiratory Medicine, Maastricht University Medical Centre, Maastricht, Netherlands; 7 Department of Pulmonology, Hospital de Santa Marta, Lisbon, Portugal; 8 Discipline of Physiotherapy, Faculty of Medicine and Health, The University of Sydney, Sydney, Australia; 9 Department of Family Medicine, Faculty of Medicine & Health Sciences, University Putra Malaysia, Serdang, Selangor, Malaysia; 10 Department of Respiratory Medicine, University of Leicester, Leicester, United Kingdom; 11 Department of Pediatric Pulmonology and Allergology, University Hospital Necker-Enfants Malades, AP-HP, Paris, France; 12 Pulmonary Diseases Clinic, Ankara Gölbaşı State Hospital, Ankara, Turkey; 13 Hospital Outbreak Support Team (HOST), H.uni network, Brussels, Belgium; 14 Department of Rehabilitation & Sports Science, Bournemouth University, Bournemouth, England, United Kingdom; 15 Faculty of Medicine, University of Zurich, Zurich, Switzerland; 16 Respiratory Research and Rehabilitation Laboratory (Lab3R), School of Health Sciences (ESSUA), University of Aveiro, Aveiro, Portugal; 17 Pulmonary Institute, Schneider Children’s Medical Center of Israel, Petach Tikvah, Israel; 18 Pulmonology Department, General Hospital of Thessaloniki, Thessaloniki, Greece; 19 Department of Primary Care and Public Health, School of Public Health, Faculty of Medicine, Imperial College London, London, United Kingdom; 20 Centre for Physical Activity Research, Copenhagen University Hospital, Copenhagen, Denmark; 21 Department of Social Medicine, School of Medicine, University of Crete, Crete, Greece; 22 School of Rehabilitation Sciences, Faculty of Health Sciences, McMaster University, Hamilton, Ontario, Canada; 23 Department of Internal Medicine II—Pulmonology Section, Asklepios Klinik Altona, Hamburg, Germany; 24 Centre for Human and Applied Physiological Sciences (CHAPS), King’s College London, London, United Kingdom; 25 Department of Paediatrics: Child and Youth Health, The University of Auckland School of Medicine, Grafton, Auckland, New Zealand; 26 The Norwegian Sports Medicine Centre, Oslo, Norway; 27 Respiratory Division, The George Institute for Global Health, Sydney, Australia; 28 School of Pharmacy, Faculty of Medical and Health Sciences, The University of Auckland, Auckland, New Zealand; Freelance medical research and writing, UNITED KINGDOM OF GREAT BRITAIN AND NORTHERN IRELAND

## Abstract

The value of ‘data-enabled’, digital healthcare is evolving rapidly, as demonstrated in the COVID-19 pandemic, and its successful implementation remains complex and challenging. Harmonisation (within/between healthcare systems) of infrastructure and implementation strategies has the potential to promote safe, equitable and accessible digital healthcare, but guidance for implementation is lacking. Using respiratory technologies as an example, our scoping review process will capture and review the published research between 12^th^ December 2013 to 12^th^ December 2023. Following standard methodology (Arksey and O’Malley), we will search for studies published in ten databases: MEDLINE, EMBASE, CINAHL, PsycINFO, Cochrane Library, Web of Science, Scopus, IEEE Xplore, CABI Global Health, and WHO Medicus. Our search strategy will use the terms: digital health, respiratory conditions, and implementation. Using Covidence, screening of abstracts and full texts will be undertaken by two independent reviewers, with conflicts resolved by a third reviewer. Data will be extracted into a pilot-tested data extraction table for charting, summarising and reporting the results. We will conduct stakeholder meetings throughout to discuss the themes emerging from implementation studies and support interpretation of findings in the light of their experience within their own networks and organisations. The findings will inform the future work within the ERS CONNECT clinical research collaboration and contribute to policy statements to promote a harmonised framework for digital transformation of respiratory healthcare.

## Introduction

Chronic respiratory diseases affect more than 545 million people worldwide, and have significant impact on individuals’ quality of life and are a major burden to healthcare systems [1]. Digital health tools can contribute to regular monitoring of respiratory conditions prone to exacerbations, supporting better health outcomes by enabling timely interventions. Conventionally, e-health and m-health have been defined as the utilisation of electronic means, such as the Information and Communication Technologies (ICT), or mobile devices and remote sensors to deliver health services [2, 3]. Digital therapeutics has been defined as “*evidence-based therapeutic interventions that are driven by high quality software programs to treat*, *manage*, *or prevent a disease or disorder*” [4]. Digital respiratory health is an umbrella term for all these modalities as well as data-enabled technologies such as artificial intelligence that have the potential to support patients with respiratory conditions in routine clinical care [5]. These technologies can support diagnosis, underpin personalised medicine, and enable self-management. By integrating environmental data and using artificial intelligence, they advise on actions to prevent exacerbations and hospitalisations. Routine data collected from these tools aids interpretation of medical images and public health decisions [6]. The COVID-19 pandemic has accelerated their implementation, showcasing their potential for rapid evaluation and deployment [7, 8] in contrast to the traditional lengthy development timeline of evaluating and implementing novel interventions [9, 10].

However, integrating digital health technologies into routine care is complex and challenging. Short-term success does not guarantee long-term sustainability. Challenges include gaining acceptance and trust from patients and clinicians, ensuring reliable communication infrastructure, addressing inequities, and navigating data privacy, security, and regulatory concerns [6, 11, 12]. Even within the European Union (EU), there is a shortfall in harmonisation across borders and sectors in the execution frameworks [2]. This deficit in harmonisation obstructs the digital transformation of healthcare, preventing it from providing safe, fair, and accessible care for everyone [13]. In Europe, this is being tackled by the European Health Data Space, which has been greeted by the European Respiratory Society (ERS) as an opportunity to push forward healthcare, research and policy-making [14, 15].

As described by the World Health Organization (WHO), digital health is a key component of achieving universal health coverage [16]. The utilisation and implementation of technological advances to improve patient outcomes and promote patient-centred care is endorsed by the EU, WHO and a top priority of the ERS [11, 17]. Only a minority of digital health studies, however progress beyond the pilot or local intervention stage [18]. The CONNECT Clinical Research Collaboration (CRC) was launched by the ERS in 2023 with the goal of addressing the over-arching challenges of whole systems implementation of respiratory digital healthcare in real-world medical settings across Europe and more widely [19]. To inform the broader objectives of the CONNECT CRC, this scoping review will systematically identify published initiatives that have embedded digital respiratory technologies in routine clinical practice, in order to describe the technology used, the implementation strategies employed, the barriers faced, and the outcomes achieved.

## Methods

### Design

Our review follows the five steps outlined by Arksey and O’Malley in their methodological framework for designing a scoping review to ensure an explicit approach to conducting the review and allow for the identification of all relevant literature, thus producing in-depth and broad results [20]. As described by Levac [21], we will include the additional step of consultation with stakeholders to help ground the work in routine practice and provide useful insights into applicability of findings. We will use the PRISMSA ScR checklist to ensure the reporting of our findings is transparent and comprehensive [22] (S1 Appendix). PROSPERO does not accept registration of scoping reviews, so our review will be registered on the CONNECT CRC website with reference to the published protocol.

### Stage 1: Identifying the research question

The review aim was discussed at a CONNECT CRC meeting at the ERS Congress 2023, and the questions refined in discussion with multidisciplinary volunteers from the CONNECT network (including clinicians, researchers, and industry from 83 countries) in a video meeting and by email. S1 Fig shows the world map of CONNECT network as of August 2024.

Our finalised research questions are:

1. What are the characteristics of respiratory digital health that have been implemented in routine clinical practice in the last 10 years?
2. What frameworks were used to develop and evaluate the implementation strategies?
3. What population level outcomes were used in the evaluation?
4. What strategies were used, and which barriers and enablers to implementation were identified?
5. What (if any) insights were described relevant to the CONNECT overarching themes of reducing inequity, enhancing patient/professional relationships, supporting the patient journey, and reducing adverse environmental impact?

### Stage 2: Identifying relevant studies

We adopted the PICO (Population, Intervention, Comparison, and Outcome) framework to define the search strategy for the scoping review. Some of the broader concepts required explanation in order for them to be operationalised during the selection process. We are using Covidence software to manage the studies for this review, from identification and de-duplication through to screening and data extraction [23]. We developed search strategies with support from a librarian and consulted previously published reviews for their key search terms [24, 25]. We used search terms for “respiratory condition” AND “digital technology” AND “implementation”. See S1 Table for the exemplar search strategies used in MEDLINE, which utilises comprehensive keywords and subheading structures; and S2 Table for the exemplar search strategies used in CABI, which utilises a simple search engine structure. The data range was from 2013–2023. We limited to the last decade because of the rapid evolution of digital healthcare and the recent acceleration of implementation with the COVID-19 pandemic. We therefore anticipated that older studies would be less relevant. We did not exclude by language. CONNECT is a global network with members fluent in most major languages.

A forward search will be performed on included studies using the International Statistical Institute Proceedings [26]. The reference lists of all included studies will be scrutinised to identify possible additional studies. We have also set up a cloud data repository for CONNECT network collaborators to record any relevant published studies that they know of through their professional and organisation networks, and the conferences they have attended. Suggested papers will be checked against the final list of included studies to ensure none are missed.

### Stage 3: Study selection

Covidence automatically removes duplicates from multiple searches, during the importing process. Volunteers from the CONNECT network who expressed interest in contributing to the study selection, were invited to attend a two stage online training programme comprising an initial training session to explain the inclusion/exclusion criteria and processes, a pilot test screening of approximately 100 titles and abstracts followed a week later by a ‘troubleshooting’ (Q&A) session where discrepancies were discussed, questions answered and agreement reached on conventions to operationalise the inclusion/exclusion criteria for the remaining studies. Following training, each study is being independently screened by two reviewers randomly selected by Covidence from the twenty-seven volunteers. This process is being closely observed by lead researchers (CYH and AHYC) who are monitoring the performance of the individual volunteer reviewers and identifying any divergent decisions. Disagreements will be discussed within the core team (CYH, AC and HP) who will then direct conflict resolution within Covidence by 3 or 4 selected volunteers.

Twenty-seven volunteers are actively involved in undertaking the title and abstract screening but it is already clear that most volunteers will only contribute small numbers. We will select a sub-group of volunteers who have contributed extensively and accurately to undertake conflict resolution, full text screening, and data extraction.

We are including any digital health interventions implemented to support routine patient care. Patient care includes but is not limited to digital support for diagnosis, self-management, monitoring, medication adherence/compliance, education, psychological support, social support, remote consultation, or health professional facing interventions. For example, a decision support system that was designed to support delivery of care would be included, but an administrative system for scheduled appointments would be excluded.

Implementation studies are not always accurately indexed [27], so we have defined the key features of ‘implementation’ to guide the selection process.

- The intervention should be available to all clinically-eligible individuals (specifically not only to people who consent to the research).
- Outcomes should reflect the uptake and impact of the intervention within the population (e.g. using routine data).
- The intervention should be delivered within the existing service. Existing staff may be upskilled for the purpose, but interventions that require additional staff/resources for day-to-day delivery are unlikely to be sustained beyond the end of the project and therefore excluded.
- We did not specify a duration for the evaluation, but only included studies in which the intervention was (or was intended to be) embedded for the long term in routine care.

We are including studies involving patients with any respiratory conditions (short or long-term) of all ages. Where there is doubt about whether a condition is ‘respiratory’, we are including those conditions covered by the Assemblies and Groups of the ERS. If there is a comparator, it is likely to be usual care, but we expect many studies to be ‘before and after’ observational designs.

We are excluding:

- Digital health interventions that do not directly involve patient care (e.g. interventions such as workflow managements, appointments or triage systems with exclusively administrative purposes).
- Health professional education (e.g. online conferences, online courses).
- Population level initiatives to manage the COVID-19 pandemic (such as contact tracing, vaccination programmes). However, COVID-19 is a respiratory disease so we are including studies investigating initiatives at an individual level (for example, ‘hospital at home’ or management of COVID-19 in people with CRD).
- Grey literature, unpublished interventions, conference abstracts and reviews. We are tagging potentially relevant abstracts and will check for a subsequent publication. Potentially useful papers that do not meet the inclusion criteria (e.g. systematic reviews) will be retrieved for background literature and reference lists checked for relevant publications.

Conflict resolution will be undertaken by a small team of five reviewers who made the largest contributions to the review process in terms of number of titles and abstracts reviewed and availability to engage in the conflict resolution discussions with CYH and AHYC. The reviewers will be trained in a similar two-stage process to perform the full text reviews, followed by conflict resolution. CYH and AHYC will oversee quality control.

### Stage 4: Data charting, extraction and management

The data will be extracted manually in Covidence. We will develop and pilot a data extraction sheet to record the data that we require to answer the five research questions.

1. Characteristics of the respiratory digital health implemented in the included study (citation, intervention, targeted population and their respiratory condition(s), country/healthcare system)
2. The frameworks (if any) that were used to develop and evaluate the implementation strategies. Examples of likely frameworks include the Non-Adoption, Abandonment, Scale-Up, Spread and Sustainability (NASSS), Normalisation Process Theory (NPT), Reach, Effectiveness, Adoption, Implementation and Maintenance (RE-AIM) framework, Behavioural Intervention Technology (BIT) model and reporting standards (StaRI), Expert Recommendations for Implementing Change (ERIC) and Theoretical Domains Framework (TDF) [28–34]. Key domains from the most commonly used frameworks will be collated and used to tailor the data extraction form.
3. Population level outcomes used in the evaluation.
4. Implementation strategies used and identified barriers and enablers.
5. Insights (if any) relevant to the CONNECT overarching themes of reducing inequity, enhancing patient/professional relationships, supporting the patient journey, and reducing adverse environmental impact.

Volunteers (n≈4) who have contributed substantially to the review process will extract data independently for two or three studies to pilot the form and as a training exercise. Following this pilot process the extraction form will be discussed within the core team (CYH, CYP, HP) and revised for use with the rest of the studies. CYH and AHYC will oversee the data extraction process, resolve conflicts between the volunteers and check the accuracy of the data prior to charting.

We will contact the corresponding authors of the included studies for missing information. If no response is received within two weeks after the first contact; we will recontact the author once, as well as the lead or senior author of the publication, depending on who the initial contact was. If we do not receive a response within a week, we will report the data as "missing" in the publication.

### Stage 5: Collating, summarising and reporting the results

We will use a PRISMA diagram to report the review process and the number of studies in each stage. As appropriate to answer the five research questions, a narrative summary of qualitative data will be collated with the charted results from the quantitative data. More specifically, we will:

1. Describe the studies’ characteristics in a table and collate the types of digital health implemented.
2. Describe the implementation frameworks used in the included studies and, if a specific framework is not used explicitly, report the domains of most commonly used frameworks that were assessed, potentially illustrating findings graphically to highlight the outcomes that are used (or not).
3. Tabulate the outcomes assessed, with a primary focus on the population-level implementation outcomes used in quantitative studies.
4. Use thematic analysis to synthesise qualitative data and authors’ observations on strategies used, and barriers and enablers to implementation identified by the authors. Use thematic analysis to synthesise qualitative data and authors’ observations on insights that address the CONNECT over-arching themes.

We will consider categorising the studies into subgroups, such as digital health before/during/after the COVID period; settings such as high, middle, low resource settings, or disease area.

### Stage 6: Consultation exercise

We will engage with a network of colleagues from the CONNECT collaboration (i.e. who expressed interest in the review, but did not contribute to screening or specific review tasks and are thus not authors), and maintain ongoing interaction with them throughout the review process. Their roles include joining stakeholder panels to advise on interpretation of findings and suggesting any potentially relevant papers of which they were aware. This group consists of academic researchers and clinicians who have experience or interest in implementing real-life respiratory digital health as well as volunteers with a technology background. They represent both high-income countries and Low- and Middle-Income Countries (LMICs), and we will invite them to contribute to online video workshops in which we will explore themes from the review and request feedback from their diverse perspectives. As appropriate, we will use digital interactive tools to facilitate interaction.

## Discussion

This review will provide an overview of the available evidence on the implementation of digital respiratory interventions. We will explore their characteristics, as well as the barriers and enablers for successful implementation which will inform future deployment of digital technologies and inform research in this area.

The findings extracted from the review will inform guidance on standardised approaches to developing, evaluating and reporting the implementation of respiratory digital health, and support harmonisation of the digital respiratory data to be collected, processed, and analysed at the patient, system and treatment level.

We will publish our findings in a peer-reviewed journal following the reporting standards for scoping reviews (PRISMA ScR) and disseminate to conferences, newsletters, and social media through our stakeholders’ networks [35]. Our findings will also be made available on the ERS website.

There are some limitations. We are aware of the poor indexing of implementation research, although we have kept our search terms broad, some relevant publications could be missed. We opted to not search the grey literature so digital respiratory implementations not published in peer-reviewed journals or conferences will not be included in this review. This was a pragmatic decision. Our preliminary searches suggested we would identify a large number of titles for screening, and to search for grey literature in all languages globally would be impractical in this minimally funded review. There was also concern about quality issues in unpublished literature. We have set a 10-year time limit for our search as we want to ensure our review reflects current technology and its implementation but we will miss reports of earlier initiatives, though these may be less relevant due to the rapid evolution of technology. We chose to include implementation studies in both LMICs and high-Income Countries. We believed this to be important, but the diverse levels of digital maturity across the world, means that the studies included in the review may be very heterogeneous. If this is the case, we will consider grouping by World bank classification of income. It is possible that some studies from LMICs will be missed if they are not published online.

## Conclusion

This scoping review, a key objective of the ERS CRC CONNECT project, will summarise and identify the scope of digital respiratory innovations and the frameworks they use to support successful implementation in routine care. The findings will be used to develop policy statements and guidance on the standardised approaches to developing, evaluating, and reporting the implementation of digital healthcare, thereby supporting a global harmonised approach to digital respiratory data at the patient, treatment, and system level to deliver safe, equitable and accessible care for all.

## Supporting information

S1 Appendix Preferred Reporting Items for Systematic reviews and Meta-Analyses extension for Scoping Reviews (PRISMA-ScR) checklist. (DOCX)

S1 Fig The world map of the CONNECT network. (TIF)

S1 Table Sample search terms on MEDLINE. (DOCX)

S2 Table Sample search terms on CABI. (DOCX)
