# Correction: Implementing digital respiratory technologies for people with respiratory conditions: A protocol for a scoping review

**DOI:** 10.1371/journal.pone.0321228

**Published:** 2025-03-27

**Authors:** Chi Yan Hui, Kathleena Condon, Shailesh Kolekar, Nicola Roberts, Katherina Bernadette Sreter, Sami O. Simons, Carlos Figueiredo, Zoe McKeough, Hani Salim, Aleksandra Gawlik-Lipinski, Apolline Gonsard, Ayşe Önal Aral, Anna Vanoverschelde, Matthew Armstrong, Dario Kohlbrenner, Cátia Paixão, Patrick Stafler, Efthymia Papadopoulou, Adrian Paul Rabe, Milan Mohammad, Izolde Bouloukaki, Shirley Quach, Malek Chaabouni, Georgios Kaltsakas, Kate Loveys, Tonje Reier-Nilsen, Anthony Paulo Sunjaya, Paul Robinson, Hilary Pinnock, Amy Hai Yan Chan

There are errors in the author affiliations. The correct affiliations are as follows:

Chi Yan Hui^1^, Kathleena Condon^2^, Shailesh Kolekar^3^, Nicola Roberts^4^, Katherina Bernadette Sreter^5^, Sami O. Simons^6^, Carlos Figueiredo^7^, Zoe McKeough^8^, Hani Salim^9^, Aleksandra Gawlik-Lipinski^10^, Apolline Gonsard^11^, Ayşe Önal Aral^12^, Anna Vanoverschelde^13^, Matthew Armstrong^14^, Dario Kohlbrenner^15^, Cátia Paixão^16^, Patrick Stafler^17^, Efthymia Papadopoulou^18^, Adrian Paul Rabe^19^, Milan Mohammad^20^, Izolde Bouloukaki^21^, Shirley Quach^22^, Malek Chaabouni^23^, Georgios Kaltsakas^24^, Kate Loveys^25^, Tonje Reier-Nilsen^26^, Anthony Paulo Sunjaya^27^, Paul Robinson^2^, Hilary Pinnock^1^, Amy Hai Yan Chan^28^

**1** Allergy and Respiratory Research Group, Usher Institute, The University of Edinburgh, Edinburgh, United Kingdom, **2** Child Health Research Centre, The University of Queensland, Brisbane, Queensland, Australia, **3** Department of Respiratory Medicine, Zealand University Roskilde Hospital, Institute of Clinical Medicine Copenhagen University, Copenhagen, Denmark, **4** School of Health and Social Care, Edinburgh Napier University, Edinburgh, United Kingdom, **5** Department of Pulmonology, University Hospital Centre "Sestre Milosrdnice", Zagreb, Croatia, **6** Department of Respiratory Medicine, Maastricht University Medical Centre, Maastricht, Netherlands, **7** Department of Pulmonology, Hospital de Santa Marta, Lisbon, Portugal, **8** Discipline of Physiotherapy, Faculty of Medicine and Health, The University of Sydney, Sydney, Australia, **9** Department of Family Medicine, Faculty of Medicine & Health Sciences, University Putra Malaysia, Serdang, Selangor, Malaysia, **10** Department of Respiratory Medicine, University of Leicester, Leicester, United Kingdom, **11** Department of Pediatric Pulmonology and Allergology, University Hospital Necker-Enfants Malades, APHP, Paris, France, **12** Pulmonary Diseases Clinic, Ankara Go¨lbaşı State Hospital, Ankara, Turkey, **13** Hospital Outbreak Support Team (HOST), H.uni network, Brussels, Belgium, **14** Department of Rehabilitation & Sports Science, Bournemouth University, Bournemouth, England, United Kingdom, **15** Faculty of Medicine, University of Zurich, Zurich, Switzerland, **16** Respiratory Research and Rehabilitation Laboratory (Lab3R), School of Health Sciences (ESSUA), University of Aveiro, Aveiro, Portugal, **17** Pulmonary Institute, Schneider Children’s Medical Center of Israel, Petach Tikvah, Israel, **18** Pulmonology Department, General Hospital of Thessaloniki, Thessaloniki, Greece, **19** Department of Primary Care and Public Health, School of Public Health, Faculty of Medicine, Imperial College London, London, United Kingdom, **20** Centre for Physical Activity Research, Copenhagen University Hospital, Copenhagen, Denmark, **21** Department of Social Medicine, School of Medicine, University of Crete, Crete, Greece, **22** School of Rehabilitation Sciences, Faculty of Health Sciences, McMaster University, Hamilton, Ontario, Canada, **23** Department of Internal Medicine II—Pulmonology Section, Asklepios Klinik Altona, Hamburg, Germany, **24** Centre for Human and Applied Physiological Sciences (CHAPS), King’s College London, London, United Kingdom, **25** Department of Paediatrics: Child and Youth Health, The University of Auckland School of Medicine, Grafton, Auckland, New Zealand, **26** The Norwegian Sports Medicine Centre, Oslo, Norway, **27** Respiratory Division, The George Institute for Global Health, Sydney, Australia, **28** School of Pharmacy, Faculty of Medical and Health Sciences, The University of Auckland, Auckland, New Zealand.
