# Supplementary figures and images for: Implementing digital respiratory technologies for people with respiratory conditions: A protocol for a scoping review
Source: PLoS One. 2024 Dec 27;19(12):e0314914. doi: 10.1371/journal.pone.0314914 (PMC11676949; doi:10.1371/journal.pone.0314914)

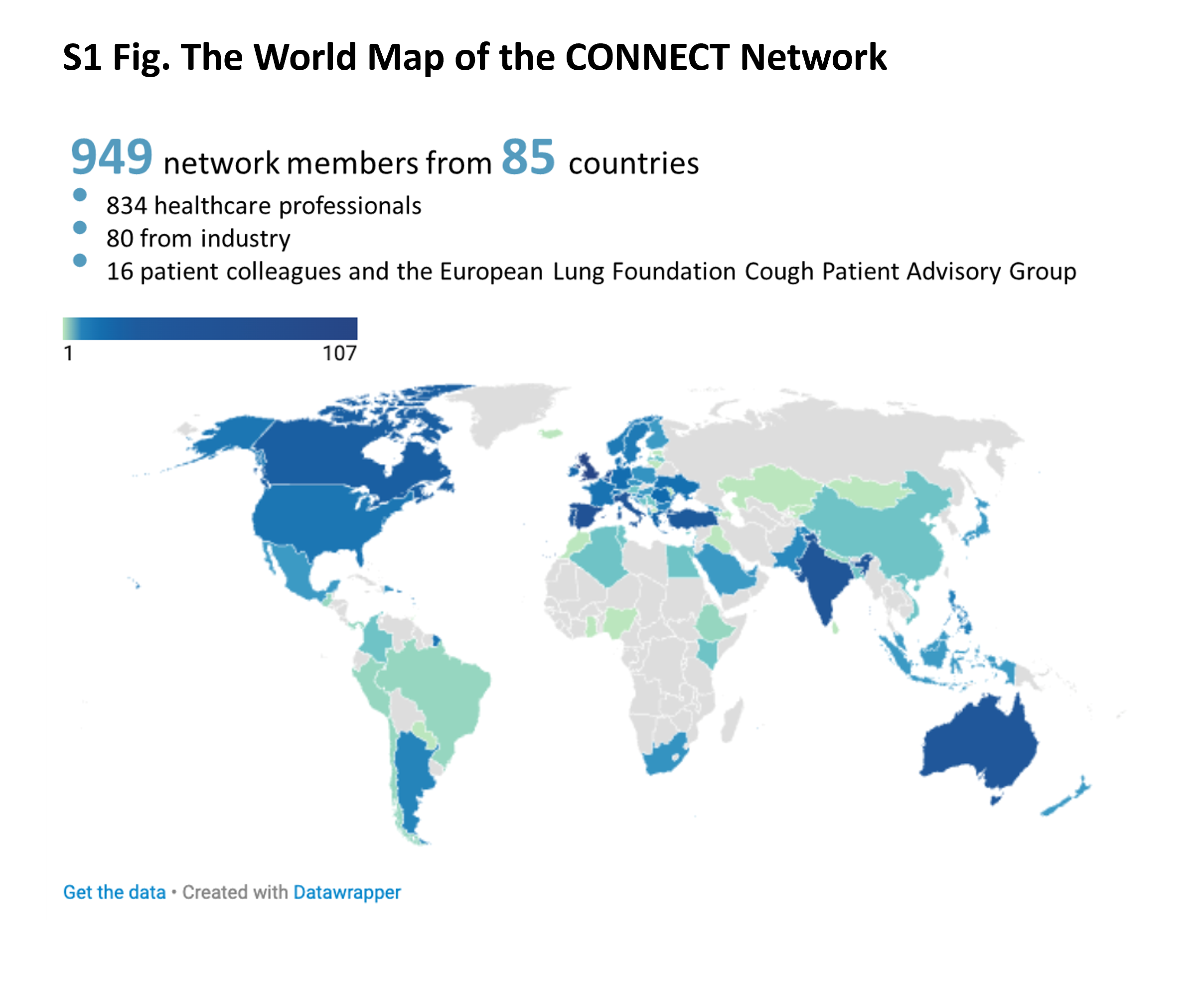

Supplement: S1 Fig — (TIF) [file pone.0314914.s002.tif]
