# Supplementary material for: Implementing digital respiratory technologies for people with respiratory conditions: A protocol for a scoping review
Source: PLoS One. 2024 Dec 27;19(12):e0314914. doi: 10.1371/journal.pone.0314914 (PMC11676949; doi:10.1371/journal.pone.0314914)
Supplement: S1 Table — (DOCX) [file pone.0314914.s003.docx]

S2 Table: Sample search terms on MEDLINE

| **MEDLINE Keywords*** |
| --- |
| **Technology search terms**  1 (Telemedicine or telecare or telehealth).ti,ab,kw.  2 (ehealth or e-health or mhealth or m-health).ti,ab,kw.  3 digital transformat*.ti,ab,kw.  4 digital strateg*.ti,ab,kw.  5 digital polic*.ti,ab,kw.  6 digital health.ti,ab,kw.  7 (smartphone* or smart-phone*).ti,ab,kw.  8 ((cell* or mobile*) adj3 phone*).ti,ab,kw.  9 (mobile adj3 tablet$1).ti,ab,kw.  10 (portable adj1 device$1).ti,ab,kw.  11 (portable adj1 sensor$1).ti,ab,kw.  12 gadget$1.ti,ab,kw.  13 (smart adj1 device$1).ti,ab,kw.  14 (smart adj1 sensor$1).ti,ab,kw.  15 (smart inhaler* or digital inhaler* or "Electronic Monitoring Device*").ti,ab,kw.  16 ((smart adj3 peak flow meter*) or spirometer*).ti,ab,kw.  17 (handheld* or hand-held*).ti,ab,kw.  18 wearable*.ti,ab,kw.  19 ("In vitro diagnostic*" adj3 device*).ti,ab,kw.  20 (automat* adj3 (phone* or telephone* or call* or system*)).ti,ab,kw.  21 (app* adj3 (smartphone* or smart-phone or mobile* or phone* or tablet* or computer*)).ti,ab,kw.  22 (internet* or web*).ti,ab,kw.  23 (sms or mms).ti,ab,kw.  24 ((text* or short*) adj3 messag*).ti,ab,kw.  25 Texting.ti,ab,kw.  26 ((electronic* adj3 (patient* or health or medic*) adj3 record*) or "EPR" or "EHR" or "EMR").ti,ab,kw.  27 (reminder adj3 (text* or system* or messag*)).ti,ab,kw.  28 (alert* adj (system or device)).ti,ab,kw.  29 ((internet adj1 of adj1 thing*) or "iot").ti,ab,kw.  30 ("virtual reality" or "VR").ti,ab,kw.  31 "augmented reality".ti,ab,kw.  32 "mixed reality".ti,ab,kw.  33 "eXtended reality".ti,ab,kw.  34 "artificial intelligence".ti,ab,kw.  35 "machine learning".ti,ab,kw.  36 "interactive voice response".ti,ab,kw.  37 chatbot.ti,ab,kw.  38 "digital twin".ti,ab,kw.  39 "blockchain".ti,ab,kw.  **Respiratory condition search terms**  40 respir*.ti,kw.  41 (chronic$ adj3 (lung$ or respiratory$ or pulmonary$)).ti,kw.  42 ((chronic* or obstruct*) adj3 (pulmonary or lung* or airway* or airflow* or bronch* or respirat*)).ti,kw.  43 (interstitial$ adj3 (lung$ or disease$ or pneumon$)).ti,kw.  44 ((pulmonary$ or lung$ or alveoli$) adj3 (fibros$ or fibrot$)).ti,kw.  45 (idiopathic and pulmonary and fibrosis).ti,kw.  46 ((pulmonary$ or lung$) adj3 (sarcoid$ or granulom$)).ti,kw.  47 (asthma* or wheez*).ti,kw.  48 emphysema*.ti,kw.  49 (COPD or COAD or COBD or AECOPD or AECB).ti,kw.  50 Bronchi*.ti,kw.  51 (cystic* adj3 fibros*).ti,kw.  52 (interstitial* adj3 (lung* or disease* or pneumon*)).ti,kw.  53 ((pulmonary* or lung* or alveoli*) adj3 (fibros* or fibrot*)).ti,kw.  54 (pneumoconiosis or silicosis).ti,kw.  55 (pulmonary adj3 eosinophi*).ti,kw.  56 (pulmonary adj2 hypertensi*).ti,kw.  57 (pulmonary adj3 sarcoid*).ti,kw.  58 sleep adj3 apnoea*.ti,kw.  59 ((lung* or pulmonary) adj3 (cancer or tumor or tumour)).ti,kw.  60 (tuberculosis or "TB").ti,kw.  61 (extrinsic adj3 allergic adj3 alveolitis).ti,kw.  62 (pneumonia or "respiratory infection").ti,kw.  63 (COVID or nCoV or SARS or MERS).ti,kw.  **Implementation search terms**  64 (real-world or "real world").ti,ab,kw.  65 ((evidence-based or "evidence based") adj1 trial$1).ti,ab,kw.  66 ((evidence-based or "evidence based") adj1 stud$3).ti,ab,kw.  67 ((evidence-based or "evidence based") adj1 intervention$1).ti,ab,kw.  68 ((real-world or "real world") adj1 (trial* or stud* or research or intervention*)).ti,ab,kw.  69 ((real-world or "real world") adj1 (setting* or practice or context*)).ti,ab,kw.  70 ((routine or normal) adj1 (setting* or practice or care or context*)).ti,ab,kw.  71 implement*.ti,kw.  72 (re-aim or reaim or CFIR or "consolidated framework for implementation research" or ("NASSS" adj1 framework*) or "Normalisation Process Theory" or "Standards for Reporting Implementation Studies").ti,ab,kw.  73 adopt*.ti,kw.  73 deploy*.ti.kw |
| **Note*** |
| We include subheadings related to telemedicine, respiratory condition, and implementation, for example, exp Telemedicine/ exp Smartphone/ exp Cell Phone/ exp Internet/exp respiratory tract diseases/ or exp bronchial diseases/ or exp lung diseases/ or exp lung diseases, fungal/ or exp lung diseases, interstitial/ or exp lung diseases, obstructive/ or exp pulmonary disease, exp "diffusion of innovation"/ or exp implementation science/ or exp technology transfer/exp Program Evaluation/exp health plan implementation/ or exp technology assessment, biomedical/ |
