# Supplementary material for: Implementing digital respiratory technologies for people with respiratory conditions: A protocol for a scoping review
Source: PLoS One. 2024 Dec 27;19(12):e0314914. doi: 10.1371/journal.pone.0314914 (PMC11676949; doi:10.1371/journal.pone.0314914)
Supplement: S2 Table — (DOCX) [file pone.0314914.s004.docx]

S3 Table: Sample search terms on CABI

| **CABI library** |
| --- |
| **Technology search terms**  telemedicine OR telecare OR telehealth OR ehealth OR mhealth OR "digital health"  "smartphone app" OR internet* OR "SMS" OR "MMS" OR "text messaging" OR “cell phone” OR “”telephone”  "electronic patient record" OR "electronic health record" OR "electronic medical record"  "smart inhaler" OR "digital inhaler" OR "Electronic Monitoring Device"  "artificial intelligence" OR "handheld device" OR "wearable device"  **Respiratory condition search terms**  respir*  chronic* lung OR chronic* respiratory OR chronic* pulmonary  COVID OR nCoV OR SARS OR MERS  **Implementation search terms**  "real world" OR "routine setting" OR “routine practice" OR "routine care" OR "routine context" OR "re-aim" OR "reaim" OR "cfir" OR "consolidated framework for implementation research" OR "nasss" OR "normalisation process theory" OR "standards for reporting implementation studies" |
